# Best Practices and Recommendations for Research Using Virtual Real-Time Data Collection: Protocol for Virtual Data Collection Studies

**DOI:** 10.2196/53790

**Published:** 2024-05-14

**Authors:** Jasmin Sanchez, Amanda Trofholz, Jerica M Berge

**Affiliations:** 1 Department of Family Medicine and Community Health, University of Minnesota Minneapolis, MN United States; 2 Department of Family Medicine and Adult and Child Center for Outcomes Research and Delivery Science University of Colorado Anschutz Medical Campus Aurora, CO United States

**Keywords:** real-time data collection, remote research, virtual data collection, virtual research protocol, virtual research visits

## Abstract

**Background:**

The COVID-19 pandemic and the subsequent need for social distancing required the immediate pivoting of research modalities. Research that had previously been conducted in person had to pivot to remote data collection. Researchers had to develop data collection protocols that could be conducted remotely with limited or no evidence to guide the process. Therefore, the use of web-based platforms to conduct real-time research visits surged despite the lack of evidence backing these novel approaches.

**Objective:**

This paper aims to review the remote or virtual research protocols that have been used in the past 10 years, gather existing best practices, and propose recommendations for continuing to use virtual real-time methods when appropriate.

**Methods:**

Articles (n=22) published from 2013 to June 2023 were reviewed and analyzed to understand how researchers conducted virtual research that implemented real-time protocols. “Real-time” was defined as data collection with a participant through a live medium where a participant and research staff could talk to each other back and forth in the moment. We excluded studies for the following reasons: (1) studies that collected participant or patient measures for the sole purpose of engaging in a clinical encounter; (2) studies that solely conducted qualitative interview data collection; (3) studies that conducted virtual data collection such as surveys or self-report measures that had no interaction with research staff; (4) studies that described research interventions but did not involve the collection of data through a web-based platform; (5) studies that were reviews or not original research; (6) studies that described research protocols and did not include actual data collection; and (7) studies that did not collect data in real time, focused on telehealth or telemedicine, and were exclusively intended for medical and not research purposes.

**Results:**

Findings from studies conducted both before and during the COVID-19 pandemic suggest that many types of data can be collected virtually in real time. Results and best practice recommendations from the current protocol review will be used in the design and implementation of a substudy to provide more evidence for virtual real-time data collection over the next year.

**Conclusions:**

Our findings suggest that virtual real-time visits are doable across a range of participant populations and can answer a range of research questions. Recommended best practices for virtual real-time data collection include (1) providing adequate equipment for real-time data collection, (2) creating protocols and materials for research staff to facilitate or guide participants through data collection, (3) piloting data collection, (4) iteratively accepting feedback, and (5) providing instructions in multiple forms. The implementation of these best practices and recommendations for future research are further discussed in the paper.

**International Registered Report Identifier (IRRID):**

DERR1-10.2196/53790

## Introduction

### Overview

Research visits with participants are a fundamental tool for data collection across multiple disciplines. Research visits can include a wide array of activities, including the assessment of eligibility, consenting, intervention administration, and baseline and follow-up data collection. Participation in in-person research visits can often be challenging for individuals limited by distance, disability, and access to transportation. Historically, most research visits have been conducted in person, face-to-face, between study participants and study staff. However, in response to the immediate need to halt in-person visits due to the COVID-19 pandemic, researchers adapted their research protocols to include remote data collection, allowing them to continue research projects without interruption [1]. The goal of this paper is to review the virtual real-time data collection research protocols used in the past 10 years to identify and propose best practices for continuing to use virtual real-time methods when appropriate for data collection going forward.

### Research Conducted Before and During COVID-19

The body of research using remote data collection methods conducted before the COVID-19 pandemic indicates that many types of data collection were conducted remotely but not necessarily in real time. Common remote data collection methods included: online survey data collection [2], online focus groups [3], video-recorded instructions for data collection [4], self-collection of biometric data (eg, taking weight at home) [5,6], and remote qualitative interviews [7,8]. The emergence of the COVID-19 pandemic in March of 2020 resulted in the sudden need for research teams to stop in-person research and move to virtual or remote data collection options that they may have never done before. Thus, many researchers shifted their data collection protocols to use remote research protocols that could be done in real time with a participant and study team member. For many study teams, these changes to revise their data collection approach to be remote and real time came quickly with limited guidance.

Findings from studies conducted both before and during the COVID-19 pandemic suggest that many types of data can be collected virtually or remotely, and, in many cases, the same type of data can be collected in several different ways. For example, multiple research teams collected anthropometric data (eg, height, weight, and waist circumference) remotely through real-time videos [5,6,9-28], while others collected anthropometric data by using recorded videos, telephone instructions, and multiple modes of instruction to assure data quality. Clinical research studies researching long-term illnesses and diagnoses (ie, Parkinson disease, Huntington disease, autism spectrum disorder, HIV, etc) used telehealth and videoconferencing during the COVID-19 pandemic [11-13,20,24,25,29]. Additionally, studies collected biospecimen samples or anthropometric data, whereas other studies administered fitness tests or surveys. These previous studies show remote data collection was occurring before COVID-19 to a limited degree, but it is still important to learn more about how research was adapted virtually during COVID-19 because of the numerous studies that shifted remotely and engaged in virtual remote data collection.

### The Value of Using Virtual Research Moving Forward

While remote data collection may no longer be a necessity dictated by the COVID-19 pandemic, there are multiple reasons to support the continuation of virtual real-time data collection. For example, remote data collection may be more accessible to some rural and limited mobility populations with a stable internet connection [30,31]. Having to attend research visits in person poses several accessibility issues, such as transportation issues, mobility problems, and the lack of childcare. Allowing participants to engage in data collection remotely removes these barriers to participation. Additionally, remote data collection is more affordable in some circumstances, as there is no need to reimburse participants for mileage. Many remote visits are shorter in duration as compared to in-person visits, thus requiring study staff to work fewer hours and therefore lowering the cost of the study. Finally, the need for specialized equipment and on-campus office space can be reduced when research is conducted remotely, as a larger study space is not needed for in-person gatherings and participants can often use their own materials from home [8]. In many cases, remote research improves opportunities to recruit a more representative study population as accessibility (ie, location) study costs and time barriers can be reduced. Reducing study costs in terms of office space, research staff costs, and mileage reimbursement allows for the funds to be used toward higher compensation for participants or to cover more study objectives and research questions. Moving forward, identifying ways to continue high-quality virtual real-time data collection visits would be a beneficial asset to many research studies.

### Study Aims

As suggested above, some researchers have been conducting virtual data collection for several years before the pandemic; however, most research using remote data collection methods were conducted during the COVID-19 pandemic. This sudden shift in the research approach meant that much of this data collection was done with little empirical guidance. Reflecting now on the various virtual real-time data collection practices’ researchers have used enables an analysis of the benefits and drawbacks of various practices used. We can evaluate how we can use learnings from protocols and approaches that were developed before the COVID-19 pandemic and those developed in response to the pandemic to facilitate decisions we make about best practices for future remote data collection. This paper aims to (1) summarize the literature over the last decade on virtual research visit protocols involving real-time data collection; (2) evaluate current practices and methods used for virtual, real-time research visits; (3) identify gaps in the current methods used for virtual, real-time research visits; and (4) provide insight on the best practice protocols for carrying out virtual, real-time research visits. The findings from this paper will offer insights on how to best carry out remote real-time data collection and improve the access and quality of research for underserved populations, which will enable study teams to pivot to various forms of data collection as needed for various circumstances that arise in the future. Remote and virtual data collection are used interchangeably throughout this paper.

## Methods

A 10-year literature review was conducted to identify past and present studies engaging in remote real-time data collection to identify strengths and weaknesses of these approaches and inform best practices for virtual real-time data collection in the future.

### Identifying Relevant Studies: Inclusion or Exclusion

A search of the published literature was carried out to identify original research articles that collected data for research purposes in remote real-time settings. The inclusion and exclusion criteria used to determine which articles could be included in this paper are described in Textbox 1.

Every article conducting real-time virtual data collection was reviewed for inclusion and exclusion criteria. The first author (JS) systematically conducted a decade-long review by searching PubMed and Google Scholar for articles published between January 1, 2013, and June 23, 2023. The 10-year span was chosen to ensure results included articles that were from before and after the pandemic. The search was conducted from April 2023 through July 2023.

The databases were searched using Boolean operators of terms derived from three concepts: (1) remote data collection, (2) virtual real-time data collection, and (3) assessment or measurement. The following search terms were used in various combinations to identify articles: “anthropometry,” “anthropometrics,” “methods,” “telemedicine,” “telehealth,” “remote consultation,” “remote visit,” “remote research,” “live,” “real-time,” and “virtual research.” The search was limited to only articles published between 2013 and 2023.

**Inclusion and exclusion criteria.**

**Inclusion criteria**

- Original research was conducted in “real time” when research staff actively engaged in data collection with a participant through a live medium such as videoconferencing (eg, Zoom or Google Chat) or telephone, where a participant and research staff could talk to each other back and forth.

**Exclusion criteria**

- Reviews
- Research that is not original
- Research that does not collect data in real time
- Research intended exclusively for medical purposes (eg, telehealth or telemedicine or describing a clinical encounter)
- Articles that solely described research protocols and did not include actual data collection and results
- Articles collecting participant or patient measures for the sole purpose of engaging in a clinical encounter as part of a health care visit or assessment
- Research visits that were only engaging in qualitative interview data collection
- Data collection that relied solely on self-report measures (ie, without virtual or remote guidance from the study team)
- Articles that described research interventions but did not involve the collection of data via a web-based platform.

### Study Selection

The selected studies underwent 3 levels of review. First, the lead author (JS) independently reviewed each article for inclusion. Articles were labeled for potential relevance as “yes,” “maybe,” or “no” based on eligibility criteria. Then, an independent reviewer also reviewed the articles and worked with the lead author to make final decisions on which articles should be included based on the previously described eligibility criteria. Third, the additional 2 coauthors (AT and JB) reviewed the studies selected after the initial reduction of studies. At each level of review, the authors reviewed article inclusion and exclusion criteria before excluding an article. A total of 22 unique articles met the eligibility criteria for inclusion in the current review (see Multimedia Appendix 1 [5,6,9-28]). Some articles (n=7) focused on collecting anthropometric data. This review included articles with study populations of different genders, different dyad types (ie, parent-child, participant-partner, and 2 adults), different racial or ethnic identities (ie, White, Black, Hispanic or Latinx, etc), and different medical conditions and abilities (ie, cancer, Huntington disease, autism spectrum disorder, HIV, and Parkinson disease). The articles involved data collection on child participants (n=9), individuals with Parkinson disease (n=4), and Huntington disease (n=1), cancer survivors (n=3), individuals with autism spectrum disorder (n=1), and adults with HIV (n=1). A total of 11 articles reported collecting data on samples that were predominantly college-educated. A total of 19 articles were composed of majority White samples, with 12 articles on White women and 7 articles on White men. One article collected anthropometric data and corrected this data in an effort to make it more accurate, using an equation that was developed using previous data [16].

### Data Collection

The data were independently extracted by the first author (JS) and reviewed by an independent reviewer. The following information was extracted from eligible studies: participant demographic data, study variables, study methods (ie, remote measurement variables), study findings, recommendations, study mode, the level of instructions provided (ie, real-time, recorded, written, etc), the level of interaction between study staff and participants, technology used to guide data collection, equipment used, accessibility features enabled, and the reason for virtual data collection. The extracted data were organized and reviewed to identify best practices. Multimedia Appendix 1 shows a detailed description of all the collected articles.

## Results

Results from the protocol review will be summarized under the themes of: (1) “modality of data collection,” (2) “equipment for data collection,” (3) “guided visit instruction or materials,” (4) “participant-staff interaction,” and (5) “reliability and validity of measures” (see Multimedia Appendix 2 [8-10,17,21-24,27-29,32]). Results and best practice recommendations from the current protocol review will be used in the design and implementation of a substudy to provide more evidence for virtual real-time data collection. We will examine the concordance between virtual real-time collection of height, weight, and neck circumference collected by participants while guided by research team members and in-person collection of height, weight, and neck circumference collected by a research team member on a sample of over 600 families. These results will add more concrete evidence to virtual real-time data collection methods that were assessed in this protocol review. We expect to carry out this substudy over the next year.

## Discussion

### Overview

This study aimed to review the different protocols and approaches used to conduct virtual real-time data collection visits over the past 10 years and during COVID-19 to identify best practices in virtual real-time data collection. Our review identified four areas in which there were both similarities and differences across studies using remote real-time data collection: (1) mode, (2) equipment, (3) guided visit instruction or materials, and (4) participant-staff interaction. We found that remote data collection approaches were used successfully across a range of different populations. A variety of approaches were used to collect data and maintain participant engagement, depending on populations and the type of data collection.

Several studies reported that providing as much equipment as possible within budget constraints resulted in successful data collection and a more representative study population. For example, multiple studies explained that providing hotspots was particularly effective in reducing barriers to participation, especially for racially or ethnically diverse and low-income populations [5,6,14,26]. Other studies that did not provide hotspots cited a lack of internet as an exclusion factor as well as a study limitation [11,15,21,23,24,28]. Studies that excluded participants without access to the internet limited the generalizability of their study population. Providing hotspots also allowed for the inclusion of rural [9] and low-income populations, for whom reliable internet may not be as readily accessible. The mode of instruction and level of interaction between staff, researchers, and participants were also emphasized as important considerations across studies. Several studies suggested that providing real-time instructions through videoconferencing was particularly helpful for specific types of data collection; in particular, real-time instructions were helpful for obtaining height and weight data as researchers could see the way participants were measuring themselves and provide real-time corrections to participants approaches to self-measurement with the goal of improving the reliability and validity of these measures.

### Recommendations

Based on findings from this review, we propose some best practice protocol recommendations to move the field forward regarding virtual real-time data collection (see Multimedia Appendix 1).

#### Create Protocols and Materials for Research Staff to Facilitate or Guide Participants Through Data Collection

Protocols and materials should be created for research staff to facilitate and guide participants as much as possible through data collection. Protocols should be as detailed as possible to ensure research staff are able to provide the same detailed instructions to all participants. While remote research removes some barriers to data collection, it also makes data collection more difficult for certain measures (ie, height); therefore, research staff should go through a thorough training process and certification so they are able to provide the same level of instruction to all participants. All materials (scales, electrocardiogram equipment, spirometers, etc) necessary to adhere to the research protocols should also be provided to reduce bias among participant results and to improve participation across diverse populations for increased generalizability of study findings. Troubleshooting guides should be provided for research staff so they are able to help participants through any issues (ie, videoconferencing not working) that may arise.

#### Provide all Necessary Equipment to Participants

Researchers should aim to provide as much necessary equipment (eg, web cameras and tablets) as possible to participants to ensure all participants have access to the same research equipment. For example, hotspots should be provided to those without access to a stable internet connection (low-income participants, rural participants, etc) so that all interested participants can fully engage in data collection. Providing scales and tape measures to participants is also beneficial, as previous studies have found results differ when using home equipment [33]. More complex studies, such as those involving electrocardiogram equipment, should provide the equipment, as this is not easily accessible to participants. It may be best to include the various types of equipment in a “toolkit” so that participants have all their needed equipment, including directions, in the same packages. Given that providing the equipment necessary to participants at their home address is costly, researchers should budget for these materials and shipping expenses when requesting funding packages.

One other key consideration that researchers need to address when conducting remote real-time data collection is exclusion criteria based on equipment requirements. For example, our review of the literature revealed that some studies chose to exclude participants that did not have reliable internet access or access to a device that could connect to the internet [5,21]. This was done because having internet services and access to internet-capable devices was deemed necessary to conduct research at a distance. However, requiring participants to have access to reliable internet and internet-capable devices can impact how representative the study population is of the population of interest.

#### Pilot Data Collection

Collecting pilot data from actual participants before starting official data collection should be strongly considered. Having a shorter pilot previsit before the official data collection visit allows participants to become familiar with the protocols and videoconferencing software, troubleshoot any technological issues, and build rapport with the research staff before data collection. This can also be an opportunity to ask for feedback from participants.

#### Iteratively Accept Feedback

Researchers should accept feedback from participants in an iterative manner that allows for adaptations of study protocols as the study advances. Accepting feedback results in meeting the needs of participants better and increases the likelihood of adherence to the research protocols.

#### Provide Instructions for Participants in Multiple Formats

Instructions for participants should be provided in multiple forms (ie, oral, written, and video instructions) to assure that all different styles of learning are accounted for and that participants understand the protocol and can look back at the directions if any questions arise. Additionally, across studies, the more that staff interacted with participants, the better the data collection visit went regarding flow and quality of data collected. This is particularly true for certain types of data collection, such as height and weight, where research staff can offer real-time corrections and guidance as the data are being collected. Textbox 2 shows best practices for virtual data collection.

**Recommended best practices for virtual data collection.**

**Proposed best practice protocol recommendations for virtual real-time data collection**

- Create protocols and materials for research staff to facilitate or guide participants through data collection.
  As many details as possible should be provided through protocols, infographics, and simplified figures or visuals to ensure research staff can provide detailed instructions to participants for successful data collection.
  Research staff should undergo thorough training and a certification process.
  Provide troubleshooting guides for research staff.
- Provide adequate equipment for participants.
  Technology necessary for research should be provided (ie, hotspots, tablets or internet-capable devices, and web cameras).
  Complex equipment that is not easily found at home should be provided (ie, electrocardiogram equipment).
- Pilot data collection
  Have a short pilot visit before data collection to troubleshoot and familiarize participants with protocols.
  Ask for participants’ feedback during pilot visits.
- Iteratively accept feedback.
  Accept feedback iteratively and adapt study protocols as the study advances.
- Provide instructions in multiple forms to participants.
  Provide instructions in multiple forms (ie, oral, written, and video instructions) to meet differing learning styles.
  The more interactive the research staff can be, the easier it is for participants to follow directions successfully.

### Strengths and Limitations

This review had both strengths and limitations. This is the first review that we are aware of that synthesizes studies using remote or virtual real-time data collection to inform best practices for future virtual data collection. There are also limitations to consider. One limitation was that studies conducting virtual or remote visits were not representative of the general population. The studies reviewed were composed of a majority of White, highly educated individuals with above-median incomes. Many studies reported using strict inclusion and exclusion criteria (ie, access to internet capable devices, access to high-speed internet, ability to use technology, and ability to download apps), which prevented individuals without access to these requirements from participating. Considering that the strict requirements to participate in research involved materials that were costly, we can assume that participants with lower incomes were not always included in these studies. Thus, we know that the recommendations listed work well for White, highly educated, and economically stable populations, but we cannot say for certain that they work for the general population. These recommendations can be used as starting points when developing accessible research, but future research should aim to include a more representative sample of the US population. Recommendations need to be created using a population that is representative of the general population to expand accessibility. Additionally, not all the methods used were validated against gold standard approaches (see Multimedia Appendix 1). Therefore, it is uncertain whether these research methods are valid and reliable. Researchers need to compare virtual methodology and measurements to gold-standard approaches to confirm that virtual research is valid and reliable.

### Conclusions

Our findings suggest that virtual real-time data collection visits are doable across a range of participant populations and to answer a range of research questions. Top considerations that emerged while reviewing the literature were that researchers should keep in mind how staff will engage with participants throughout virtual visits, the importance of creating materials and protocols that will facilitate measurement collection, piloting protocols ahead of time, encouraging feedback from participants throughout data collection, and that inclusion or exclusion criteria needed to be balanced to ensure a representative population of participants can fully engage in remote data collection protocols. Recommended best practice protocols were developed based on these identified considerations: (1) creating protocols and materials for research staff to facilitate or guide participants through data, (2) providing adequate equipment, (3) piloting data collection, (4) iteratively accepting feedback, and (5) providing instructions in multiple forms.

## Appendix

Multimedia Appendix 1 Description of articles reviewed by population, measurement type and approach, and feasibility.

Multimedia Appendix 2 Themes on the logistics of collecting data from participants remotely.

## Data Availability

The data sets generated during and analyzed during this study are available from the corresponding author on reasonable request.
