# Supplementary material for: Best Practices and Recommendations for Research Using Virtual Real-Time Data Collection: Protocol for Virtual Data Collection Studies
Source: JMIR Res Protoc. 2024 May 14;13:e53790. doi: 10.2196/53790 (PMC11134243; doi:10.2196/53790)
Supplement: Multimedia Appendix 1 [file resprot_v13i1e53790_app1.docx]

| **Citation** | **Population** | **Measures collected virtually** | **Who took measurements** | **Engagement with study staff** | **Visual instructions** | **Equipment** | **Findings** |
| --- | --- | --- | --- | --- | --- | --- | --- |
| Forseth B, et al (2021) [9]^a^ | Rural Children and their caregivers (child mean age = 8.3 yrs. old, caregiver mean age = 38.2, n = 38 rural families) | Height and weight | Parent | Staff guided and corrected participants | Mailed instructions and Instructional video | Participants were mailed instructions and any equipment that they did not have (e.g., scale, tape measure) prior to their data collection visit. | Families were able to successfully collect their own height and weight measurements yielding relatively accurate results. |
| Hoenemeyer TW et al (2021) [10]^a^ | Cancer survivors and their partners (mean age = 60) | Weight, waist circumference | Cancer survivors and their partners measured each other | Staff guided and corrected participants | Mailed instructions and Instructional video | Participants were sent a package of remote assessment materials along with a step-by-step instruction booklet of procedures. |  |
| Dorsey ER et al (2015) [11] | Adults with Parkinson’s disease (mean age: 62) (n=166) | Patient diagnostic data, assessment of motor skills | Participant | Staff guided and corrected participants | Mailed instructions | Prior to visit, participants were mailed written materials. The study provided a web camera and technical support if needed. | Providers were able to successfully characterize and validate self-reported diagnosis in adults with Parkinson’s disease. |
| Dorsey ER et al (2015) [12] | Adults with Parkinson’s disease (mean age: 63.6) (n=52) | Patient diagnostic data, assessment of motor skills | Participant | Staff guided and corrected participants. | Mailed instructions | Prior to visit, participants were mailed written materials. The study provided a web camera and technical support if needed. A test connection visit was conducted prior to the data collection visit. | Providers were able to successfully characterize and validate self-reported diagnosis in adults with Parkinson’s disease. |
| Buro A et al (2021) [13] | Children with autism spectrum disorder at risk for obesity (age = 12-21, n = 27) | Height and weight | Participant | Staff guided and corrected participants | Mailed lesson booklet and lesson manual | Prior to the visit, participants were sent a lesson booklet and lesson manual with a ruler and scale. | Obtaining virtual height and weight of people with autism is feasible |
| Tabachnick AR et al (2021) [14] | Mothers and their infants (mean age = 30 and 44 weeks, n = 54 total) | Behavioral and psychophysiological data | Mother of infant | Staff guided and corrected participants | Visual instructions were provided in paper form | Prior to the visits, participants were provided with ECG equipment, tablets, mobile hotspots, and a virtual assessment kit with all the necessary materials needed for data collection. | Participants can collect valid behavioral and psychophysiological data when being guided by research staff. |
| Vales C et al (2021) [15] | Children (age = 5-6, n = 58) | Semantic differentiation effects | Child | Staff explained directions and observed children completing the task | Instructional video was provided | Participants were expected to have a computer and internet access | Conceptual replication of two semantic differentiation effects can be feasibly conducted virtually while yielding valid data. |
| Datar A et al (2023) [16] | Adolescents (mean age = 17, n = 216) | height and weight, Installation County Obesity Rate (InstaCOR), Time preference | Participant | Staff guided and corrected participants | None provided | Prior to visit, participants were mailed measurement equipment | Collected height and weight was valid and comparable to gold standard data collection when measurements were guided by research staff and corrected after. |
| Ghosh-Dastidar M et al (2020) [17]^a^ | Children and their parents (age 13-58, n = 20) | Height, weight, waist circumference | Participant | Staff guided and corrected participants | None provided | Participants were provided with a stadiometer, scale, and measuring tape. | Very small differences (<0.03) were found between virtual and in-person height, weight, and waist circumference measurements. |
| Bove R et al (2022) [18]^a^ | Adults (age = 18-65, n = 100) | Neurostatus and Neurological evaluation (Neurostatus NS-EDSS) | Participant | Neurologist administered assessment virtually | None provided | Prior to the visit, a vision card and red piece of paper was sent to participants. | Data collection was feasible and found to be lower cost for both researchers and participants. |
| Tarolli CG et al (2020) [28]^a^ | Adults with atypical Parkinsonian syndrome (mean age = 65.7, n = 45) | Motor function assessments, Patient diagnostic data | Participant | Neurologist and evaluator administered assessment virtually | None provided | Prior to the visit, participants were sent a portable blood pressure cuff and a web camera if needed. | There was excellent concordance between participant and investigator diagnosis. |
| Bull MT et al (2014) [23]^a^ | Adults with Huntington's disease (mean age = 56.5, n = 13) | Motor assessments (MoCA) | Participant | Physician administered assessment virtually | None provided | Surveys and the written portion of the MoCA were sent by mail | Virtual visits conducting motor assessment for people with Huntington's disease were found to be feasible and reliable. |
| Tenenbaum A et al (2021) [21]^a^ | Children in Israel (age - 3 -18, n = 107) | Height and Weight | Child's Parent | Staff guided and corrected participants | Mailed instructions and Instructional video | Prior to the visit participants were sent written instructions and a video. | In-person and virtually collected height and weight were found to be comparable. |
| Policarpo S et al (2021) [19] | Adults with HIV (mean age = 54.6, n = 112) | Weight, waist circumference, physical activity | Participant | Staff guided and corrected participants | None provided | Participants used equipment they had at home | Measurements worked for study purposes. |
| Lavín-Pérez AM et al (2023) [20]^a^ | Adults (mean age = 58.48, n = 40) | Senior Fitness test | Participant | Staff guided and corrected participants | None provided | Camera, chair, stopwatch, pen/ruler, unclear if these were sent to participants | Study results found no difference (p> 0.05) between in person and virtually collected measures. |
| Nelson PM et al (2021) [22]^a^ | Children and their parents (mean child age = 4.77, mean parent age = 36.59, n = 132 | Standardized and experimental cognitive assessments | Child Parent | Staff and experimenters administered assessment virtually | None provided | Parent-child dyads without an electronic device were mailed an Amazon Fire table | No reliable differences were found between tests. |
| Vogel EA et al (2022) [5] (BREATHE study; footnote: this manuscript described two different studies) | Adults (age = 18-25, n =77) | Biospecimen, perception of timing, breathe tests | Participant | Staff guided and corrected participants | None provided | fully charged tablets with pre-paid internet access, paper questionnaires and saliva sample kits | Data collection was feasible and acceptable for tobacco research. |
| Vogel EA et al (2022) [5] (AIRS; footnote: this article covered two different studies) | Adults (age = 21-65, n = 31) | Biospecimen, perception of timing, breathe tests | Participant | Staff guided and corrected participants | None provided | Study kit was mailed with a spirometer kit, disposable mouthpieces for the spirometer, a saliva cotinine collection kit, an NELF nasal swab kit, disposable gloves, cotton balls, alcohol wipes, ice packs and three pre labelled return packages. | Data collection was feasible and acceptable for tobacco research. |
| Jensen-Roberts S et al (2022) [24] | Adults with Parkinson’s disease (mean age = 67.8, n = 277) | Parkinson's Disease Diagnosis | Participant | Staff guided and corrected participants | None provided | Participants were sent the University of Pennsylvania Smell Identification Test (UPSIT) via mail. | Providers were able to successfully characterize and validate self-reported diagnosis in adults with Parkinson’s disease. |
| Blair CK et al (2020) [25] | Cancer survivors (ages = 29-32, 47-50 months, n = 59) | Functional Mobility | Participant | Staff guided and corrected participants | Mailed instruction | Prior to the visit a test toolkit, instructions and procedures were sent to participants. | Older participants can follow instructions, use tablets, resulting in successful data collection. |
| Morini G and Blair M (2021) [26]^a^ | Children (age = 29–32-month-olds, 47–50-month-olds, n = 59) | Eye-gaze data during a word-learning task | Participant | Staff observed child | None provided | Group 1 was provided with all equipment necessary while group 2 used whatever computer screen and camera was available to them | The study found that it is feasible to collect remote eye-gaze data with young children. |
| Guidarelli C et al (2022) [27]^a^ | Cancer survivors and their partners (mean age = 62.5, n = 176) | Physical function | Participant | Staff guided and corrected participants | None provided | Participants were provided with any equipment they didn't have (e.g., armless, non-rolling, straight-backed, standard height chair; and a measuring tape ≥ 14 | Remote assessments of physical function are feasible and reliable |
| Saad B et al (2022) [6] | Syrian refugee families (n = unknown) | Biological samples | Participant | Staff were available for questions | None provided | Fully charged tablets with pre-paid internet access, paper questionnaires and saliva sample kits | It is possible to continue longitudinal research that involves the collection of biological samples remotely |

^a^These articles have data comparing in-person to virtual measurements.
