# Supplementary material for: Best Practices and Recommendations for Research Using Virtual Real-Time Data Collection: Protocol for Virtual Data Collection Studies
Source: JMIR Res Protoc. 2024 May 14;13:e53790. doi: 10.2196/53790 (PMC11134243; doi:10.2196/53790)
Supplement: Multimedia Appendix 2 [file resprot_v13i1e53790_app2.docx]

**Multimedia Appendix 2.** Themes on the logistics of collecting data from participants remotely.

### Overview

Within the articles reviewed, several consistent themes emerged surrounding the logistics of collecting data from participants remotely. Study findings have been organized around these themes and are discussed in detail below and in Multimedia Appendix 1. The themes showcase that not all studies were carried out identically and highlight how studies had numerous similarities and differences in approach based on their specific study needs. Studies with protocols and procedures that encapsulated the breadth of themes we found in the analysis tended to report better outcomes in terms of reaching their research goals through web-based platforms.

### Modality of Data Collection

A crucial part of remote research planning involves deciding the modality of data collection. Many studies reviewed reported using videoconferencing, such as Zoom (Zoom Video Communications) [32], Skype (Microsoft) [8], etc, to meet with participants in real time, face-to-face. Each study attempted to use the methods that made the most sense for their research questions. For example, some studies carried out their data collection through phone or a combination of phone and videoconferencing platforms [20,22]. Phone calls were often used before videoconferencing to obtain consent and inform participants of the study procedures. Using phone calls before using videoconferencing to obtain consent was seen as helpful as it eliminated any issues that may arise with participants who had not used web-based conference platforms.

### Equipment for Data Collection

Common equipment needs for many of the studies included web cameras, hotspots, internet-capable devices (ie, laptops, iPads, or smartphones), scales, and tape measures. Studies differed in providing participants with necessary equipment (n=20) versus relying on participants (n=2) to have their own equipment available at home for study use, with a few studies (n=6) making it optional for participants to request equipment if necessary.

Certain research questions required specific equipment, which was either provided by research staff to participants or it was expected of the participants to have these items at home (eg, a scale for measuring weight). Studies provided equipment such as web cameras, hotspots, scales for weight measurements, height tools, computers or tablets, electrocardiogram equipment, blood pressure cuffs, biospecimen collection kits, and specific measurement kits (ie, virtual assessment kits and test toolkits).

Other studies reported having to exclude participants who did not have access to required study materials such as a telephone, tablet, computer, and high-speed internet or were unwilling to download applications to their devices. The amount of equipment provided by research studies varied depending on the study design and research questions, as well as the research team’s budget.

### Guided Visit Instruction or Materials

The mode of instruction provided varied by study. Many studies (14/22, 64%) solely provided live instructions either by phone or videoconferencing. Other studies used multiple modes of instruction, including the use of written, recorded, and live instructions. Other studies used written and live instructions (n=5); a total of 3 studies used recorded, written, and live instructions; and 1 study used prerecorded and live instructions. The more complex studies were, the more instructions were provided to participants, often in multiple formats (eg, paper, written, or video) to ensure participants understood directions.

A total of 3 studies trained staff on how to provide technical support to ensure participants could connect to their various platforms. A couple of studies (n=2) had a previsit before their actual data collection visits to ensure participants knew how to use the technology and that there would not be any technical difficulties during their visit.

### Participant-Staff Interaction

The level of interaction between participants and research staff also varied across the studies reviewed. Most of the studies (16/22, 73%) guided their participants in real time, providing direction on how to conduct the various measures and collect the data during their time together. Other studies (n=2) solely observed while participants performed the measurements and did not offer guidance or correction; a total of 3 studies had professionals administer research tests on participants; and finally, 1 study had staff present research materials and be available for questions but did not provide direct guidance.

### Reliability and Validity of Measures

Half (11/22, 50%) of the studies provided reliability data comparing their remote measurements to in-person gold standard measurements, and study results suggest that collecting data (ie, height and weight, waist circumference, motor function assessments, etc) remotely is reliable and valid [9,10,17,21-24,27-29]. These studies are marked in Multimedia Appendix 1 along with their recommendations.
